# Interpretation and Expectations Among Mothers of Children with Anxiety Disorders: Associations With Maternal Anxiety Disorder

**DOI:** 10.1002/da.22211

**Published:** 2013-11-10

**Authors:** Faith Orchard, Peter J Cooper, Cathy Creswell

**Affiliations:** 1 School of Psychology and Clinical Language Sciences, University of ReadingReading, United Kingdom; 2 Department of Psychology, University of StellenboschStellenbosch, South Africa

**Keywords:** mother, child, anxiety, cognition, interpretation

## Abstract

**Background:**

Models of the development and maintenance of childhood anxiety suggest an important role for parent cognitions: that is, negative expectations of children's coping abilities lead to parenting behaviors that maintain child anxiety. The primary aims of the current study were to (1) compare expectations of child vulnerability and coping among mothers of children with anxiety disorders on the basis of whether or not mothers also had a current anxiety disorder, and (2) examine the degree to which the association between maternal anxiety disorder status and child coping expectations was mediated by how mothers interpreted ambiguous material that referred to their own experience.

**Methods:**

The association between interpretations of threat, negative emotion, and control was assessed using hypothetical ambiguous scenarios in a sample of 271 anxious and nonanxious mothers of 7- to 12-year-old children with an anxiety disorder. Mothers also rated their expectations when presented with real life challenge tasks.

**Results:**

There was a significant association between maternal anxiety disorder status and negative expectations of child coping behaviors. Mothers’ self-referent interpretations were found to mediate this relationship. Responses to ambiguous hypothetical scenarios correlated significantly with responses to real life challenge tasks.

**Conclusions:**

Treatments for childhood anxiety disorders in the context of parental anxiety disorders may benefit from the inclusion of a component to directly address parental cognitions. Some inconsistencies were found when comparing maternal expectations in response to hypothetical scenarios with real life challenges. This should be addressed in future research.

## Introduction

Anxiety disorders in childhood are common and their adverse impact is significant.[1–3] Models of the development and maintenance of childhood anxiety suggest a role for parent cognitions in which negative expectations of the child's coping abilities lead to behaviors that inadvertently maintain child anxiety.[4–6] Specifically, compared to parents of nonanxious children, parents of anxious children have been found to expect their child to perceive threat, experience negative emotions, and feel that they have little or no control when presented with ambiguous situations; and parents of such children see themselves as having less control than parents of nonanxious children over their child's responses.[7–11] These findings are important as parents’ expectations of their children's responses to challenge have been found to influence parents’ behavioral responses.[12]

Although negative expectations regarding children's responses to challenging situations are likely to reflect, to some degree, parents adjusting their expectations based on their experience of parenting a child with an anxiety disorder, parental expectations are also influenced by parental factors, including parental mental state.[13] This is of particular relevance in relation to childhood anxiety disorders as psychological disturbance, in particular anxiety disorder, is common among parents of anxious children.[14,15] Notably, the presence of such disorder in the parents of anxious children is associated with relatively poor child treatment outcomes.[16,17]

Recent evidence suggests that parental anxiety may promote negative expectations regarding child responses. Specifically, parental anxiety has been found to be associated with increased expectations that children will perceive situations as threatening and become distressed, and that parents themselves will have less control over their child's anxious responses.[6,11,18] Notably, Lester et al.[6] reported that the association between parental anxiety and interpretations regarding threat to the child was mediated by the degree to which parents’ interpreted threat in their own environment. Their research included a small number of fathers; however, the results remained the same when analyses were restricted to mothers only. This pattern of results was also replicated by Lester, Field, and Cartwright-Hatton[19] with a large sample of mothers. Although these findings require replication within clinical populations, they suggest that a more general interpretative bias may account for parents’ biased expectations about the children's responses, and that this bias may warrant attention within treatments for children with anxiety disorders in the context of parental anxiety disorders.

The current study examined interpretation and expectations among mothers of children with a diagnosed anxiety disorder. In our study, we use interpretation to refer to how mothers interpret ambiguous situations that they would experience, and expectations to refer to how the mother expects her child to respond to ambiguous scenarios which they might encounter. We set out to compare the expectations of anxious and nonanxious primary caregivers of children with an anxiety disorder, and to investigate whether differences in expectations are mediated by parent's self-referent interpretative style. As mothers are the most common primary caregivers attending our clinical service, and that there is evidence to suggest that fathers responses may be associated with child anxiety in different ways from mothers,[20] we elected to restrict our sample to primary caregiving mothers. We also sought to identify which particular cognitive constructs were associated with maternal anxiety by considering maternal expectations and interpretation relating to threat, negative emotions, and perceived control. Since age and gender are likely to influence maternal cognitions and behaviors,[21] we ensured that groups were balanced on these factors. We also took account of maternal low mood, as it is commonly comorbid with anxiety[22] and is also associated with negative maternal cognitions.[23]

The majority of studies of parents’ expectations have employed hypothetical scenarios, although a small number of studies have used naturalistic stress tasks and results have generally been consistent across methods.[9] Our final aim was, therefore, to explore the degree to which maternal expectations in response to hypothetical scenarios are consistent with maternal expectations in response to a real-life challenge task.

The following hypotheses were examined among mothers of a clinical sample of children who met criteria for a current anxiety disorder:

1. Compared with nonanxious mothers, mothers with a current anxiety disorder will expect their child to experience more threat, more negative emotions, and less perceived control in response to hypothetical ambiguous scenarios and real life challenge tasks.
2. Compared with nonanxious mothers, mothers with a current anxiety disorder will interpret situations as more threatening and will anticipate more negative emotions and less perceived control in response to self-referent hypothetical ambiguous scenarios and real life challenge tasks.
3. Maternal self-referent interpretation will mediate the association between maternal anxiety status and their child-referent expectations in response to hypothetical ambiguous scenarios and real life challenge tasks.

## Materials and Methods

### Participants

Two hundred seventy-one clinically anxious children, aged 7–12, and their mothers (all biological primary caregivers) gave informed consent and took part in the study. For 136 of the children, their mothers also fulfilled diagnostic criteria for a current anxiety disorder (ANX), and for 135 children, their mothers did not fulfill diagnostic criteria for a current anxiety disorder (NONANX). The groups were well balanced on child age, gender, ethnicity, and socioeconomic status (see Table1).

**TABLE 1:** Sample characteristics

	Maternal anxiety disorder (ANX), *N* = 136	No maternal anxiety disorder (NONANX), *N* = 135	
Age (months; mean, *SD*)	119.87 (19.33)	118.53 (19.98)	*t*(269) = .57, *P* = .57
Gender (percent female)	54%	54%	χ^2^(1) = .004, *P* = .95
Family SES percent “higher professional”	54%	64%	χ^2^(1) = 1.69, *P* = .19
Ethnicity percent white British	84%	90%	χ^2^(1) = 1.83, *P* = .18
SCAS-c total (mean, *SD*)	40.76 (18.66)	39.57 (17.89)	*t*(260) = .52, *P* = .60
SCAS-p total (mean, *SD*)	42.28 (15.89)	36.38 (17.17)	*t*(246) = 2.81, *P* = .01
SDQ-p behavioral problems (mean, *SD*)	2.78 (2.02)	2.18 (1.86)	*t*(256) = 2.47, *P* = .01
SMFQ-c (mean, *SD*)	8.59 (5.93)	7.84 (5.54)	*t*(261) = 1.06, *P* = .29
DASS-D (mean, *SD*)	11.87 (9.15)	3.80 (4.41)	*t*(174) = 8.72, *P*<.001
DASS-A (mean, *SD*)	9.10 (7.34)	2.18 (2.80)	*t*(156) = 9.70, *P*<.001^a^

ANX, mothers with current anxiety disorder; NONANX, mothers without current anxiety disorder; SCAS-c, Spence Children's Anxiety Scale-child report; SCAS-p, Spence Children's Anxiety Scale-parent report; SDQ-p, behavioral problems: Strengths and Difficulties Questionnaire-parent report (conduct problems); SMFQ-c, Short Mood and Feelings Questionnaire-child report; DASS-D/A, Depression, Anxiety and Stress Scale-depression/anxiety score; SD, standard deviation.

a Based on equal variances not assumed.

All participating children were recruited through referrals by local health and education service personnel and were assessed by graduate psychologists using the Anxiety Disorders Interview Schedule for DSM–IV: Child and Parent version[24] (ADIS-IV: C/P, see below). They were included on the basis of having an anxiety disorder as their primary diagnosis (see Table2). No difference was found between groups for child anxiety disorder by comparing primary diagnosis (χ^2^(11) = 13.14, *P* = .28). There was also no difference between groups on clinical severity ratings (CSRs) of the primary diagnosis (*t*(261) = 1.71, *P* = .09). Participating children in each group did differ on presence of an externalizing disorder (χ^2^(1) = 4.91, *P* = .03) and a trend was found on presence of a mood disorder(χ^2^(1) = 3.69, *P* = .06).

**TABLE 2:** Child diagnostic characteristics

	ANX	NONANX
Anxiety diagnosis primary (overall, %)	*N* = 136	*N* = 135
Separation anxiety disorder	28.7 (67.6)	23.0 (49.6)
Social anxiety disorder	20.6 (69.1)	21.5 (60.0)
Specific phobia	10.3 (46.3)	22.2 (42.2)
Panic disorder w/o agoraphobia	0 (1.5)	0.7 (0.7)
Panic disorder with agoraphobia	0.7 (0.7)	0.7 (0.7)
Agoraphobia w/o panic disorder	5.9 (7.4)	4.4 (7.4)
Generalized anxiety disorder	30.9 (70.6)	23.7 (56.3)
Anxiety disorder NOS	2.9 (4.4)	3.7 (3.7)

ANX, mothers with current anxiety disorder; NONANX, mothers without current anxiety disorder; w/o, without; NOS, not otherwise specified.

Mothers in the ANX group were included on the basis of having an anxiety disorder as their primary diagnosis, determined by their responses to the ADIS-IV (see below).[25] Frequencies of primary anxiety disorders and overall anxiety disorders, respectively, of mothers in this group were as follows: generalized anxiety disorder (57%, 68%), social phobia (15%, 43%), specific phobia (19%, 54%), agoraphobia without panic disorder (2%, 13%), panic disorder (1%, 2%), obsessive compulsive disorder (1%, 2%), hypochondriasis (2%, 2%), posttraumatic stress disorder (0%, 1%), and anxiety disorder not otherwise specified (3%, 4%). The NONANX group was included on the basis of not meeting criteria for a current anxiety disorder. As expected, mothers in the ANX and NONANX groups differed significantly on symptoms of anxiety (*t*(156) = 9.70, *P* < .001) and depression (*t*(174) = 8.72, *P*<.001; see Table1) using the self-report Depression, Anxiety and Stress Scales[26] (DASS-21; see below).

### Procedure

The study was approved by the Berkshire Local Research Ethics Committee and the University of Reading Research Ethics Committee.

Mothers and children completed initial diagnostic interviews and symptom questionnaires (see below). In a subsequent research assessment mothers completed ambiguous scenarios questionnaires (ASQ; see below) in a separate room from their child. They were then reunited with their child, and three challenge tasks were administered to the child and mother. The family was presented with a social challenge, then a performance challenge, and finally a physical challenge. In the social task, children were asked to give a short presentation of between 3 and 5 min in length to a video camera. For the performance task, “tangram” puzzles were used: children were asked to place geometric pieces together to form particular shapes. In the physical challenge task, children were invited to place their hands in a black box to find out what the “scary items” were inside. The box contained four fluffy or squidgy toys. Maternal expectations regarding their child's and their own responses were assessed using rating scales immediately before each task (see below).

### Measures

#### Structured Diagnostic Interviews with Children and Parents

Children were assigned diagnoses on the basis of the ADIS-C/P,[24] a structured diagnostic interview with well-established psychometric properties.[27] Where children met symptom criteria for a diagnosis (based on either child or parent report), they were assigned a CSR ranging from 0 (*complete absence of psychopathology*) to 8 (*severe psychopathology*). As is conventional, only those children who met symptom criteria with a CSR of 4 or more (*moderate psychopathology*) were considered to meet diagnostic criteria. Assessors (psychology graduates) were trained on the standard administration and scoring of the ADIS-C/P through verbal instruction, listening to assessment audio recordings and participating in diagnostic consensus discussions. The presence or absence of a current maternal anxiety disorder diagnosis was assigned on the basis of the ADIS-IV[25] that followed the same administration procedure as the ADIS-C/P. Overall reliability for the assessment team was excellent. Reliability for ADIS-C/P diagnosis was .98 (child report), .98 (mother report); and for CSR .99 (child report), .99 (mother report).^1^ Reliability for ADIS-IV diagnosis was .97.

#### Symptoms

The Spence Children's Anxiety Scale (SCAS-C/P)[28,29] was administered to assess child and parent reported symptoms of anxiety. The child version is a self-report questionnaire that requires children to rate how often they experience each of the 38 anxiety symptoms, presented alongside six positive filler items, on a 4-point scale from 0 (*never*) to 3 (*always*). The Short Mood and Feelings Questionnaire (SMFQ-C)[30] was administered to assess child-reported symptoms of low mood. The SMFQ-c is a brief, 13-item measure that requires children to report how often in the past 2 weeks they have experienced a number of symptoms on a 3-point scale from 0 (*not true*) to 2 (*certainly true*). Finally, the conduct problems scale (5 items) from the Strengths and Difficulties Questionnaire (SDQ-P)[31] was used to assess behavioral disturbance. Parents respond to each item on a 3-point scale from 0 (*not true*) to 2 (*certainly true*). The parent report version of the SDQ was used as parents are often considered to be most reliable in terms of providing information on children's externalizing symptoms.[32] The depression and anxiety scales of the DASS-21[27] were administered to all participating mothers. The DASS-21 is a brief 21-item measure that requires adults to report how much the statements apply to them from 0 (*not at all)* to 3 (*very much*). Internal consistency varied among measures with the majority being good to excellent[33] (SCAS-c α = .91; SCAS-p α = .89; SMFQ-c α = .82; SMFQ-p α = .93; SDQ-c (conduct) α = .60; SDQ-p (conduct) α = .63; DASS-A α = .80; DASS-D α = .91).

#### Hypothetical Ambiguous Scenarios (Parent and Self-Referent)

Mothers completed two versions of the adapted ASQ.[7,8] Both the parent (ASQ-p) and self-referent version(ASQ-s) comprised of 12 hypothetical situations. The parent version included situations relating to the child (e.g., “Your child arranges to have a party at 4 o'clock and by half past 4 no one has arrived”). Mothers were asked to rate (1) how their child would feel in this situation (0 = *not at all upset*; 10 = *very upset*; negative emotion), (2) how much they could change how their child feels about this (0 = *not at all*; 10 = *a lot*; perceived parent control of child feelings), (3) how much their child would be able to do about this situation (0 = *nothing*, 10 = *a lot*; perceived child control), and (d) how much they could change what their child does next time (0 = *nothing*, 10 = *a lot*; perceived parent control of child performance). They were also asked to give a free response to the question “Why will your child think this is happening?” (threat-free response), and choose which of two alternatives (threat/nonthreat) their child would be more likely to think in this situation (threat-forced choice).

The self-referent version included situations relating to the parent (e.g., “Not long after starting your new job your boss asks to see you”). Mothers were asked to rate (1) how they would feel in this situation (0 = *not at all upset*; 10 = *very upset*; negative emotion), and (2) how much they would be able to do about this situation (0 = *nothing*, 10 = *a lot*; perceived self-control). They were also asked “Why do you think this is happening?” (threat-free response), and were asked to (c) choose which of two alternatives (threat/nonthreat) they would be more likely to think in this situation (threat-forced choice).

Responses to open ended questions were coded as “threat” (e.g., “nobody wants to come to the party”) or “nonthreat” (e.g., “they must be in a traffic jam”). If answers included both threatening and nonthreatening responses, they were coded as “threat.” If “don't know” was given as an answer it was coded as “nonthreat.” Scores were totaled across the 12 scenarios. If data were missing, a total score was created using the average score multiplied by 12. If more than 25% of a participants data were missing it was excluded from analyses. A second independent coder coded a sample of the responses (*n* = 20) to assess inter-rater reliability. Intraclass correlations showed good reliability (parent ICC = .84, self-referent ICC = .89). As in previous reports,[8] the free and forced choice threat responses were combined to reduce the number of variables (parent threat *r* = .76; mother-self threat *r* = .78), as were the two questions measuring “perceived parent control”[18] (perceived parent control, *r* = .82). Internal consistency varied from acceptable to excellent for each subscale for parent and parent-self measures on the current study data (child negative emotion, α = .72; parent control of child, α = .91; child control, α = .77; child threat, α = .75;; self-referent negative emotion, α = .89; self-referent control, α = .87; self-referent threat, α = .87).

#### In Vivo Challenge Tasks: Expectations

Mothers were asked to rate (1) how their child would feel about doing the task (0 = *not scared at all*, 10 = *extremely scared*; negative emotion), (2) how well they thought their child would do the task (0 = *not well at all*, 10 = *extremely well*; threat), (3) how much their child could do about how the task went (0 = *nothing at all*, 10 = *a lot*; perceived child control), (4) how much they would be able to make a difference in their child's feelings about doing the task (0 = *not at all*, 10 = *a lot*) (perceived parent control of child feelings), and (5) how much they would be able to make a difference in how well their child did the task (0 = *not at all*, 10 = *a lot*; perceived parent control of child performance). Mothers also rated (1) how they would feel if they were doing the task (negative emotion), (2) how well they would do (threat), and (3) how much control they would have (perceived self-control). Ratings for the three separate challenges were combined in order to look at overall responses over a range of situations. To reduce the number of variables for analyses, as in the ASQ, the two questions measuring perceived parent control were combined (*r* = .76).

## Results

### Preliminary Analyses and Analytic Plan

Continuous data were screened in relation to the assumptions of parametric tests.[34] Where assumptions were violated, confirmatory analyses were conducted by running analyses with 1,000 bootstrap samples. All results were consistent, suggesting that the original analyses were robust to the violations of assumptions, so results based on the original (nonbootstrapped) analyses are presented for simplicity.

Where groups differed on potential confounding variables, we conducted sensitivity analyses, that is, without (1) participating children with a behavioral disorder (*n* = 81) and a mood disorder (*n* = 37) and (2) participating mothers with a mood disorder (*n* = 29). This approach was taken, rather than entering diagnostic or self-report scores as covariates due to concerns about colinearity.[35] The results were consistent with those which included the full sample, so data based on the full sample are reported. We did not control for differences in maternal reported child anxiety symptoms (SCAS-p) as this group difference is likely to reflect, at least in part, what we are testing in the study hypotheses. Notably, the groups did not differ significantly in terms of child-reported anxiety symptoms or clinician-rated anxiety severity (see Tables1 and 2). Due to the large sample size, some of the measures have missing data as is reflected in the differing degrees of freedom.

To examine the hypotheses, a series of multivariate analysis of variance (MANOVA) were conducted with group (maternal anxiety disorder vs. nonanxious mothers) as the independent variable, and responses to ambiguous scenarios and challenge tasks as the dependent variables. The parent-dependent variables for ambiguous scenarios and challenge tasks were expectations of child negative emotions, control, threat, and mother control of child. The mother self-dependent variables for ambiguous scenarios and challenge tasks were interpretations of mother-self negative emotions, threat, and control. The assumptions of MANOVA were met except for in the analyses of parent self-referent ambiguous scenarios and challenge tasks. In these cases, analyses were rerun using bootstrapping and results were consistent. The original results have been reported for simplicity. Furthermore, as follow-up between-subject effects were used, where findings did not withstand Bonferroni corrections, this has been reported. Associations between mothers’ responses to the ASQ-s and ASQ-p were examined using bivariate correlations. To examine the path from maternal anxiety to maternal expectations (ASQ-p) via self-interpretation (ASQ-s), we conducted indirect analyses using bootstrapping.[36] These analyses were conducted where the following criteria were met: (1) the initial variable (maternal anxiety group) is associated with the potential mediator (ASQ-s), and (2) where the potential mediator (ASQ-s) is associated with the outcome variable (ASQ-p).

To explore the degree to which hypothetical ambiguous scenarios reflect maternal expectations in response to a real-life challenge task, correlational analyses were conducted. The above analyses were then repeated using maternal expectations in challenge tasks as the dependent variables in order to assess whether the pattern of results was consistent across these two methods of assessing maternal expectations.

### Hypothesis 1

There was a significant group effect for mother's expectations of their child's responses to ambiguous scenarios (*V* = .08, *F*(4,235) = 5.12, *P* = .001; *d* = .29; Table3). Follow-up between-subject tests indicated that, compared to nonanxious mothers, mothers with anxiety disorders perceived their child as likely to have a higher level of negative emotions (*F*(1,238) = 13.27, *P*<.001; *d* = .47), feel less in control (although this did not withstand Bonferroni correction; *F*(1,238) = 4.56, *P* = .03; *d* = .28), and be more likely to perceive threat (*F*(1,238) = 16.83, *P*<.001; *d* = .53).When responses to the ASQ were compared to responses to real-life tasks, there were significant positive associations for all of the indices following Bonferroni corrections: child negative emotions (*r* = .33), child control (*r* = .35), child threat (*r* = .27), and parent control of child (*r* = .46). There was also a trend for an association between maternal anxiety status and mothers’ expectations of their children's responses to the challenge task (*V* = .03, *F*(4, 247) = 2.14, *P* = .08, *d* = .18; although as shown in Table3 there was a significant group effect for child control, but as the overall group effect was not significant, this should be viewed with caution).

**TABLE 3:** Differences between groups

	ANX, *N* = 136	NONANX, *N* = 135	
ASQ parent (mean [*SD*], range)			*F*(1, 238)
Threat	14.5 (4.0), 5–23	12.1 (4.7), 1–23	16.83, *P* <.001
Child control	46.3 (17.1), 8–94	51.2 (17.5), 15–103	4.56, *P* = .03
Parent control	114.6 (39.1), 14–219	113.5 (35.8) 24–214	0.0, *P* = .99
Negative feelings	77.6 (15.9), 24–111	70.1 (15.4), 29–103	13.27, *P* <.001
ASQ self (mean [*SD*], range)			*F*(1, 245)
Threat	11.0 (5.2), 1–24	5.3 (3.3), 0–18	106.73, *P*< .001
Control	55.4 (20.5), 12–105	67.8 (23.7), 12–115	21.05, *P*< .001
Negative feelings	66.4 (18.8), 11–116	47.5 (18.4), 4–100	74.48, *P*< .001
Challenge tasks parent (mean [*SD*], range)			*F*(1, 250)
Threat	13.8 (4.2), 5–30	13.0 (4.2), 5–28	2.23, *P* = .14
Child control	18.9 (4.5), 8–30	20.4 (4.5), 6–30	7.80, *P* = .01
Parent control	32.3 (10.8), 7–60	33.8 (9.8), 8–56	1.18, *P* = .28
Negative feelings	14.2 (5.0), 3–26	13.5 (5.1), 3–27	2.10, *P* = .15
Challenge tasks self (mean [*SD*], range)			*F*(1, 256)
Threat	17.5 (5.1), 8–30	14.1 (4.5), 5–30	34.24, *P*< .001
Control	17.6 (5.4), 5–29	21.5 (4.5), 5–30	36.87, *P*< .001
Negative feelings	14.7 (6.1), 0–29	9.8 (4.8), 0–24	52.34, *P*< .001

ANX, mothers with current anxiety disorder; NONANX, mothers without current anxiety disorder; SD, standard deviation; ASQ, ambiguous scenarios questionnaire.

### Hypothesis 2

There was a significant effect of group for mothers’ responses to self-referent ambiguous scenarios (*V* = .33, *F*(3, 243) = 40.45, *P*< .001; *d* = .81). Compared to nonanxious mothers, mothers with anxiety disorders anticipated more negative emotions (*F*(1, 245) = 74.48, *P*< .001; *d* = 1.10), perceived situations as more threatening (*F*(1, 245) = 106.73, *P*< .001; *d* = 1.31), and saw themselves as less in control (*F*(1, 245) = 21.05, *P*< .001; *d* = .58). When responses to the ASQ were compared to responses to real-life tasks, there were significant positive associations for parent-self negative emotions (*r* = .49), parent-self threat (*r* = .39), and parent-self control (*r* = .48) following Bonferroni corrections. There was also a significant effect of group on mother's expectations of their own responses in the challenge task (*V* = .20, *F*(3, 254) = 20.75, *P*< .001; *d* = .57). Follow-up between-subject tests indicated that maternal anxiety disorder status was significantly associated with increased negative emotion ratings (*F*(1, 256) = 52.34, *P*< .001; *d* = .90), increased threat ratings (*F*(1, 256) = 34.24, *P*< .001; *d* = .73), and reduced control ratings (*F*(1, 256) = 36.87, *P*< .001; *d* = .76).

### Hypothesis 3

**Figure 1:**
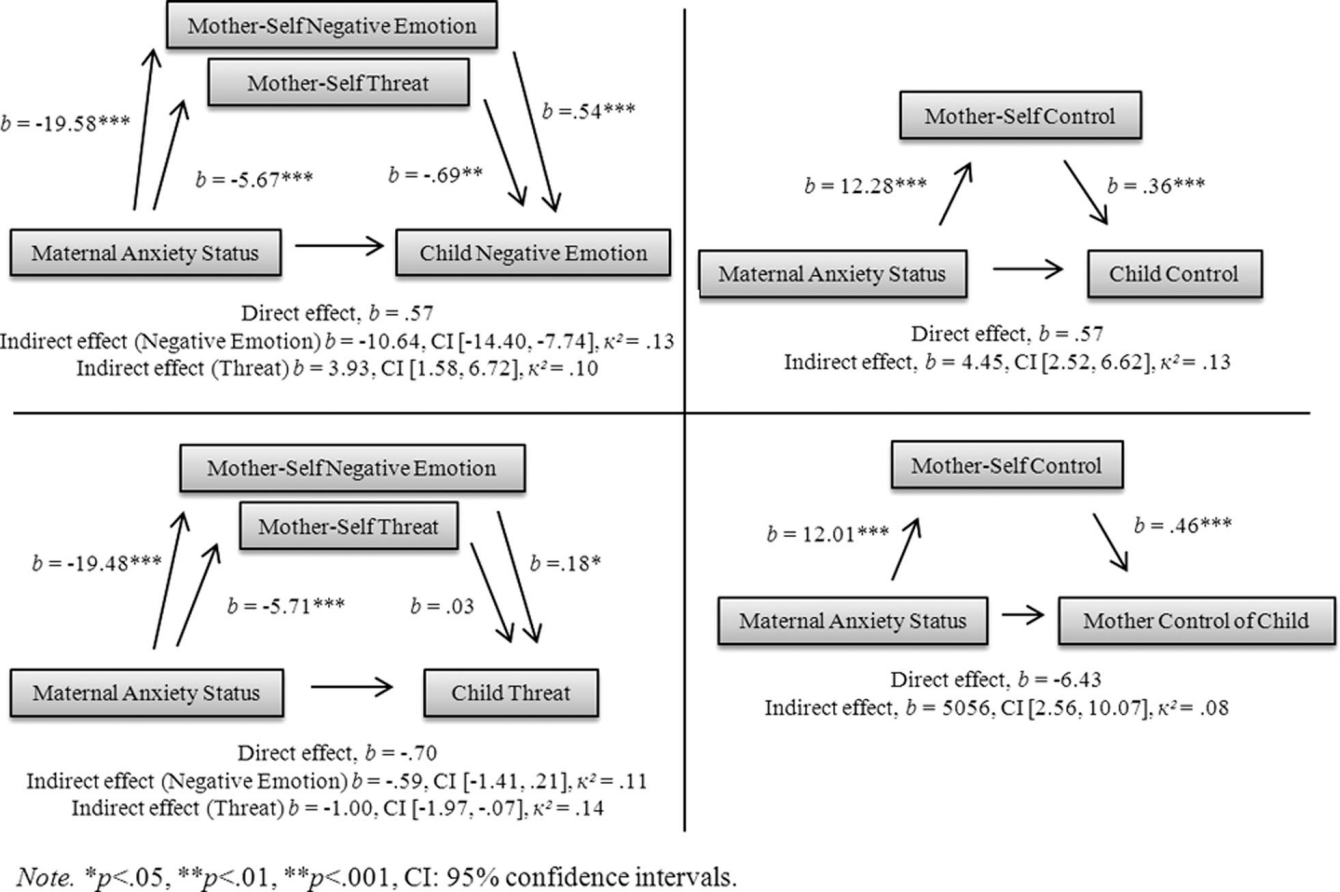
Mediation models for ambiguous scenarios task.

As shown in Table4, significant correlations were identified between responses to the parent and self-referent ASQ measures. Maternal expectations of child negative emotions were significantly associated with self-referent perception of threat (*r* = .29) and negative emotions (*r* = .57). Maternal expectations of child control were significantly associated with self-referent perception of control (*r* = .48). Maternal expectations of child perceived threat were significantly associated with self-referent perception of threat (*r* = .35) and negative emotions (*r* = .33). Finally, maternal perceived control of the child's responses was significantly associated with self-referent perception of control (*r* = .26). Mediation models can be seen in Fig.1. In summary, significant indirect associations were found between (1) maternal anxiety status and perception of child negative emotions via self-referent perception of threat and negative emotions, (2) maternal anxiety status and perception of child control via self-referent perception of control, (3) maternal anxiety status and perception of child threat via self-referent perception of threat, and (4) maternal anxiety status and perception of parent control of child via self-referent perception of control.

**TABLE 4:** Correlations between mother-self and child ambiguous scenarios questionnaires

ASQ parent (child)	ASQ parent (self) Threat	Control	Negative feelings
Threat	.35*	.10^a^	.33*
Child control	−.20	.48*	−.10
Parent control of child	.02	.26*	.16
Negative feelings	.29*	−.06	.57*

ASQ, ambiguous scenarios questionnaire.

* Significant after applying Bonferroni correction *P*<.0016.

a Bootstrapped result due to inconsistency with original analyses.

As shown in Table5, significant correlations were identified between responses to parent and self-referent responses in the challenge tasks. Maternal expectations of child negative emotions were significantly associated with self-referent negative emotions (*r* = .32). Maternal expectations of child control were significantly associated with self-referent perception of control (*r* = .42) and threat (*r* = .21). Maternal expectations of child perceived threat were significantly associated with self-referent perception of control (*r* = .21). Finally, maternal perceived control of the child's responses was significantly associated with self-referent perception of control (*r* = .40) and threat (r = .34).

**TABLE 5:** Correlations between mother-self and child challenge tasks

Challenge task parent (child)	Challenge task parent (self) Threat	Control	Negative feelings
Threat	.16	.21*	.03
Child control	.21*	.42*	−.08
Parent control of child	.34*	.40*	−.001
Negative feelings	−.04	−.08	.32*

* Significant after applying Bonferroni correction *P* < .0016.

**Figure 2:**
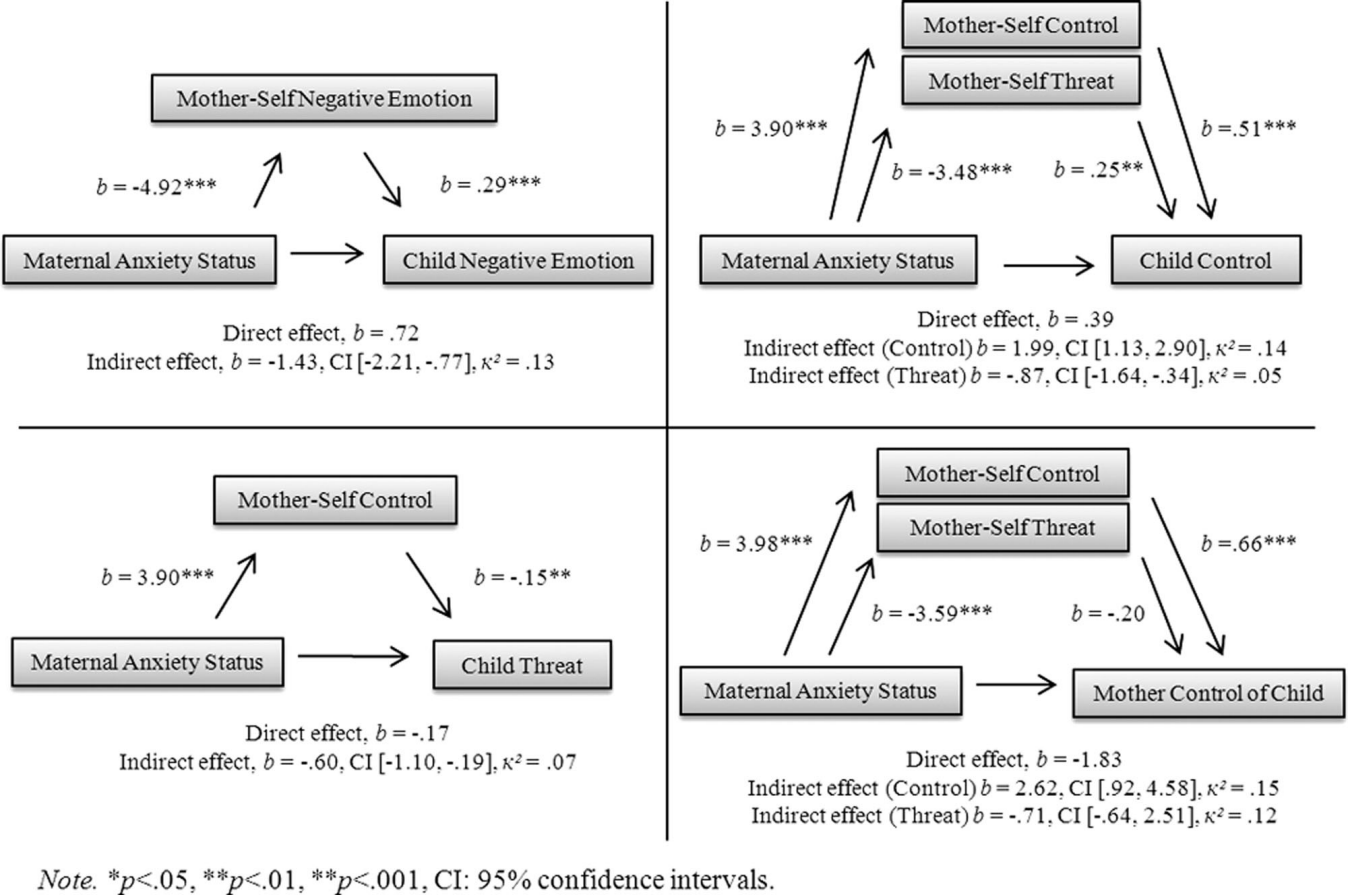
Mediation Models for challenge task.

Mediation models can be seen in Fig.2. In summary, significant indirect associations were found between (1) maternal anxiety status and perception of child negative emotions via self-referent perception of negative emotions, (2) maternal anxiety status and perception of child control via self-referent perception of control and threat, (3) maternal anxiety status and perception of child threat via self-referent perception of control, and (4) maternal anxiety status and perception of parent control of child via self-referent perception of control.

## Discussion

The primary aim of this study was to examine the association between maternal anxiety and mothers’ expectations of anxious children's responses. Compared with nonanxious mothers, mothers with an anxiety disorder expected their child to perceive situations as more threatening, to experience more negative emotions, and to be less in control in response to hypothetical ambiguous scenarios. Compared with nonanxious mothers, mothers with a current anxiety disorder also perceived themselves as experiencing less control, more negative emotions, and more threat. Finally, consistent with findings from a community study,[6,19] the associations between maternal anxiety disorder and maternal expectations of their child's response were mediated by how mothers interpreted self-referent scenarios.

We were also interested in the extent to which responses to ambiguous hypothetical scenarios correspond to mothers’ expectations when their child is confronted with real life challenge situations. Encouragingly, in the current study there were significant positive associations between responses to hypothetical scenarios and real life challenges and the same pattern of results was also found across these two methods for mother's self-referent expectations and interpretation. When child-referent expectations and interpretation were assessed, larger group differences were found when ambiguous scenarios were used than real life challenge tasks. It is possible that expectations of the real life task were less influenced by maternal anxiety as the situation was set up to be unambiguously challenging. Further research would benefit from including real life challenge tasks that incorporate a greater degree of ambiguity.

Negative expectations of children's coping abilities have been found to be associated with parental responses, such as overinvolved or intrusive behaviors,[12] that themselves have been found to increase anxiety, at least among high-trait anxious children.[37,38] The current findings suggest that, in the context of high parental anxiety, targeting parental cognitions may be important to optimize treatment outcomes for child anxiety disorders. Furthermore, the findings suggest that targeting parent's self-referent thinking styles could well have an impact on parental expectations regarding their child. This suggestion warrants examination within an experimental or treatment context. The findings also have possible implications for the intergenerational transmission of anxiety-related cognitions. Specifically, Creswell, Cooper, and Murray[39] hypothesized that parents’ interpretative biases and expectations may lead to behaviors that promote the development and maintenance of children's anxious cognitions, for example, negative expectations may lead parents to restrict child autonomy, which may promote the child's sense that the world is threatening and they are not able to cope. Further research is warranted to directly examine potential routes from parental to child anxiogenic cognitions.

Strengths of the current study were the inclusion of age- and gender-balanced groups, and the consideration of potential confounding effects (mother-reported child behavioral disturbance and maternal depression that were both elevated in the context of maternal anxiety disorder). It is important to note certain limitations, however, including the sample demographics (mostly high socioeconomic status, Caucasian families) that limit the extent to which the findings can be generalized. The study recruited primary caregivers, and we could not take parental gender into account given the low number of primary caregiving fathers attending our clinic, so we focused exclusively on mothers, which is a clear limitation.[20] We also did not counterbalance task order and therefore task-specific effects could not be examined. Finally, the cross-sectional nature of the study means that conclusions cannot be drawn with regards to the direction of the effects.

## Conclusion

Maternal expectations of anxious children's response to ambiguous and novel situations differed according to maternal anxiety status despite there being no differences in the severity of child self-reported anxiety symptoms. How mothers interpreted self-referent scenarios accounted, at least in part, for group differences in their expectations about their children's responses. The findings suggest that treatments for childhood anxiety disorders in the context of parental anxiety disorder may benefit from the specific targeting of parents’ anxious thinking styles.

## Notes

1 As different assessors interviewed the parent and child simultaneously, reliability figures for parent and child report were calculated separately.
